# Deprivation of Auditory Experience Influences Numerosity Discrimination, but Not Numerosity Estimation

**DOI:** 10.3390/brainsci12020179

**Published:** 2022-01-29

**Authors:** Alessia Tonelli, Irene Togoli, Roberto Arrighi, Monica Gori

**Affiliations:** 1 U-VIP, Unit for Visually Impaired People, Istituto Italiano di Tecnologia, 16163 Genova, Italy; monica.gori@iit.it; 2 Cognitive Neuroscience Department, International School for Advanced Studies (SISSA), 34136 Trieste, Italy; irene.togoli@gmail.com; 3 Department of Neuroscience, Psychology, Pharmacology and Child Health, University of Florence, 50121 Florence, Italy; roberto.arrighi@gmail.com

**Keywords:** numerosity perception, cross-modal calibration, deafness

## Abstract

Number sense is the ability to estimate the number of items, and it is common to many species. Despite the numerous studies dedicated to unveiling how numerosity is processed in the human brain, to date, it is not clear whether the representation of numerosity is supported by a single general mechanism or by multiple mechanisms. Since it is known that deafness entails a selective impairment in the processing of temporal information, we assessed the approximate numerical abilities of deaf individuals to disentangle these two hypotheses. We used a numerosity discrimination task (2AFC) and an estimation task, in both cases using sequential (temporal) or simultaneous (spatial) stimuli. The results showed a selective impairment of the deaf participants compared with the controls (hearing) in the temporal numerosity discrimination task, while no difference was found to discriminate spatial numerosity. Interestingly, the deaf and hearing participants did not differ in spatial or temporal numerosity estimation. Overall, our results suggest that the deficit in temporal processing induced by deafness also impacts perception in other domains such as numerosity, where sensory information is conveyed in a temporal format, which further suggests the existence of separate mechanisms subserving the processing of temporal and spatial numerosity.

## 1. Introduction

In the last few years, several studies showed that animals, including humans, possess the ability to make quick and reliable—but still approximate—estimates of the number of objects in their visual field, an ability called *number sense* [1,2]. This number sense relies on dedicated brain mechanisms tuned to the encoding numerical information, as demonstrated by neurophysiological evidence in the monkey [3,4,5,6,7] and neuroimaging results in the human brain [8,9,10,11,12].

However, it is still disputed whether the representation of numerosity is supported by single or multiple mechanisms. On the one hand, several studies suggest a generalized and amodal system for numerosity processing. For instance, it has been shown that numerosity adaptation [13], a phenomenon in which a sustained presentation of specific numerosity (i.e., adaptor stimulus) distorts the numerosity of a subsequent stimulus in a repulsive fashion (i.e., an adapted stimulus more numerous than the adaptor is perceived as even more numerous, and vice versa) and generalizes across different stimulus presentation formats. Indeed, numerosity adaptation occurs for arrays of items scattered over space and presented simultaneously (spatial numerosity), as well as for a series of visual flashes presented sequentially over time. Adaptation also distorts perceived numerosity for stimuli of different sensory modalities [14] and across the perceptual and motor domains, as shown by the phenomenon of motor adaptation [15,16,17]. All these results point to the existence of a high-level numerosity processing system that is capable of encoding information, irrespective of the low-level features of the stimuli.

However, other studies suggest the existence of multiple and partially independent brain mechanisms for the perception of different kinds of numerosity (i.e., spatial vs. temporal numerosity [18]). For example, while adaptation to the numerosity of a sequence of flashes affects the perceived numerosity of an array of dots subsequently presented around the adapter region (affecting spatial numerosity), the opposite effect (from spatial to temporal numerosity) has not been found [14]. Moreover, Cavdaroglu et al. [19,20] investigated which brain areas were activated in response to either the temporal or spatial numerosity of visual stimuli. They observed two different networks for the two features. In particular, the results showed that a common network in the occipital visual areas and the posterior parietal cortex (PPC) was selectively activated by simultaneously presented arrays of items (spatial numerosity). However, the same was not true for the sequence of flashes (temporal numerosity), raising the possibility of multiple and independent mechanisms for numerosity perception.

Regardless of whether *number sense* is mediated by a single or multiple brain mechanisms, many studies have found that quantity and other magnitude dimensions are encoded with a common metric (i.e., a similar neural code). According to the framework proposed by “A Theory of Magnitude” (ATOM [21]), numerosity is not an independent process, but it is linked to space and time in a generalized magnitude system. Indeed, it has been shown that when time, space, and numerosity are modulated together, they can influence each other [22,23,24]. Moreover, time and numerosity might share similar accumulation mechanisms [25], and each system is organized along a continuum in a spatial fashion [2,26]. Whereas the numerosity dimension could be considered a supramodal attribute, the same does not apply to spatial and temporal information. Indeed, different sensory modalities may affect the reliability of the encoding of these two domains. For instance, vision is the most accurate sense for the processing of spatial information [27], while audition has a higher sensitivity for the processing of temporal information [28].

According to the cross-sensory calibration theory [28,29,30,31,32], during development, the sensory modality that has the highest sensitivity in a specific domain calibrates the other senses. Namely, during development, vision calibrates audition in spatial representation [33], while audition tunes vision to encode temporal information [28,34]. Consequently, the lack of one sensory modality during the early stages of life would prevent the other senses from benefiting from such an inter-sensory calibration and induce sensitivity impairments. Several studies have indeed shown that deaf individuals present impairments in processing temporal information of both visual and tactile stimuli within the second as well as the millisecond ranges [35,36,37,38,39,40,41,42]. For example, in the study by Amadeo et al. [35], the participants were tested in a temporal bisection task in which they had to judge which between two temporal intervals was shorter. The authors observed that the performance of the deaf group was poorer compared with the hearing controls. This impairment in the temporal representation observed in deaf individuals is likely to reflect the auditory system’s lack of calibration over the visual and tactile systems in the early stages of life. It is possible to hypothesize that this decreased sensitivity in processing temporal information in the deaf may also have consequences in other domains of perception, such as that of numerosity.

In the present study, we assess the ability of early deaf and hearing participants to estimate or discriminate both temporal (i.e., stimuli presented over time) and spatial (i.e., dots presented over space) numerosity to test two hypotheses. First, taking into account the relation between numerical and temporal processing within a common magnitude system [21], it might be that an impairment in the processing of one dimension might result in impaired perception of another dimension. More specifically, if temporal perception is impaired—as in the case of deaf individuals—numerosity perception might be impaired too. Alternatively, this impairment might be selective to aspects of numerosity perception more closely related to temporal processing, such as in the case of numerical information conveyed by stimuli presented over time (i.e., sequential numerosity) but not when the same numerical information is delivered by arrays of dots (i.e., simultaneous). Addressing these points will provide evidence about the nature of the number sense, specifically whether numerical perception is supported by a unique mechanism or by separate mechanisms related to different representational formats (i.e., spatial versus temporal numerosity). Furthermore, we decided to test our hypotheses using two different tasks: a discrimination task and an estimation task. The first type of task allows us to assess numerosity perception in relative terms, as it entails a comparison between two stimuli, while the estimation task involves judgements of numerosity in absolute terms.

## 2. Materials and Methods

### 2.1. Participants

We tested a different number of deaf and hearing participants for each task and condition (for more details, see Table 1). Each deaf participant was randomly assigned to the task and numerosity presentation format to have an equally sized sample among the tasks and conditions.

To decide on the sample number, we performed power analysis based on the results of Amadeo et al. [35]. Specifically, this analysis was aimed at determining the sample size needed to achieve a meaningful interaction between groups and conditions in the discrimination task using linear mixed model analysis. First, we simulated a data frame using the average and standard deviation of the conditions independent and coherent for each group (deaf and control). Then we created a corresponding model fitted with “*lme4*” 1.1–21 package in R, with GROUP (deaf and hearing participants) and STIMULI (temporal and spatial numerosity) as fixed effects, while participants were added as a random effect. To select the appropriate sample size for our experiment, we simulated the power for different sample sizes using the “*mixedpower*” ver 2.0 package in R [43]. We explored the power for a sample size of 10, 15, 20, and 25, running 2000 simulations with an alpha err. prob of 0.5. We considered acceptable a sample size with a power of 0.8. The results of this analysis showed that a sample between 10 and 15 participants was sufficient to achieve a significant interaction (see Supplementary Materials). Moreover, we also took into account the expected availability of early deaf participants during the testing of this experiment (approximately 6 months) (i.e., the amount of early deaf individuals that we could realistically expect to recruit during the course of the project).

Deaf participants were recruited at the National Association for Deaf (Ente Nazionale per la protezione e assistenza dei Sordi) in Genova, Italy. All participants reported no history of neurological or cognitive deficits, and they had normal or corrected-to-normal vision (for more details, see Table 2). All deaf participants had bilateral severe to profound hearing loss in both ears, and all lost their hearing before 5 years of age (see Table 1 for details). None of the deaf participants received a cochlear implant. The research protocol was approved by the ethics committee of the local health service (Comitato Etico, ASL3 Genovese, Italy) (Comitato Etico Regione Liguria, Genoa, Italy; Prot. IIT_UVIP_COMP_2019 N. 02/2020, 4 July 2020) and conducted according to the Declaration of Helsinki. All participants gave written informed consent before starting the test.

The majority of the deaf participants that took part in the present experiment already participated in a previous study of our lab, in which they had been tested in a temporal bisection task that revealed significant impairment in processing temporal information [35] (for more information, see Supplementary Materials).

### 2.2. Apparatus and Stimuli

Visual stimuli were generated using the Psychophysics Toolbox in MATLAB (version 3.0.15, The Mathworks, Inc., Natick, MA, USA). Stimuli were presented on a CRT monitor (Barco—model CID 421Calibrator Line, Barco, Inc., Kwun Tong, Hong Kong) with a resolution of 1280 × 1024, corresponding to 41° × 31.2° for the visual angle from a viewing distance of about 57 cm and with a refresh rate of 100 Hz. The background of the screen was always gray (RGB: 125 125 125). The experiment included discrimination and an estimation task tested in separate sessions. In both tasks, two sets of stimuli were used, defining two experimental conditions. In one condition, we used stimuli presented sequentially (temporal numerosity), whereas in the other condition, we used stimuli presented simultaneously over a region of space (spatial numerosity). Sequential stimuli were structured as a series of briefly presented white discs with a diameter of 10°. In each sequence, individual stimuli were presented for 40 ms each, with a variable inter-stimulus interval (ISI) depending on the stimuli numerosity, with only the constraint of the minimum ISI lasting as a single impulse (40 ms). The overall duration of an entire sequence was 2 s. One sequential stimulus was presented in each trial in the estimation task, with numerosity ranging from 3 to 20 flashes. In the discrimination task, we presented two sequences for each trial: a reference sequence always including 12 flashes, while the numerosity of the test sequence was determined adaptively by a QUEST routine on a trial-by-trial basis. QUEST is an adaptative algorithm that uses a Bayesian approach to set the new test numerosity using all the information available from previous trials, supplemented by prior knowledge from the literature and previous experiments [44]. The numerosity range was set between 4 and 24.

The simultaneous stimuli were arrays of black and white dots (50% proportion) randomly scattered across a circular area with a diameter equal to 14°. The diameter of each dot was 0.4°. The gap among the dots was 1.3 times their size. Each dot array stimulus was presented for 500 ms. Again, in the estimation task, we presented a single stimulus in each trial, with numerosity ranging from 3 to 20 dots. The numerosities of 3 and 4 were used as catch trials. In the discrimination task, we again presented a reference and a test stimulus in each trial. The reference’s numerosity was kept constant at 12 dots, while the numerosity of the test was determined in each trial by the adaptive QUEST routine.

Data and study materials (code) will be available upon request to the corresponding author.

### 2.3. Procedure

The experiment was performed in a quiet and dimly lit room with participants sitting in front of the screen at a distance of about 57 cm. In all conditions, the participants were required to keep their eyes on the central fixation point throughout the experiment.

Before starting the experiment, the participants performed five practice trials (not included in the analysis) to familiarize themselves with the task. When testing the hearing group, the experimenter verbally explained the task, while in the case of deaf individuals, an interpreter explained the task using Italian sign language (LIS).

#### 2.3.1. Discrimination Task

**Figure 1:**
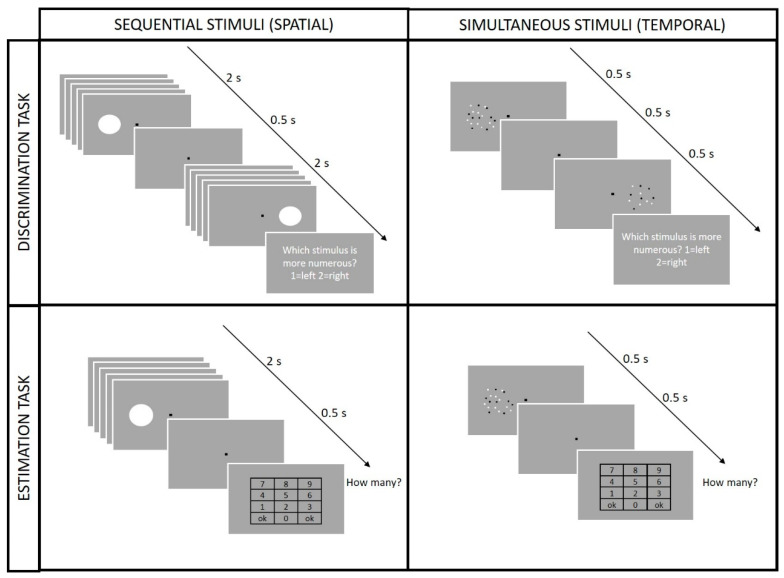
Schematic representation of the different experimental conditions. Participants performed either a discrimination task (**upper** panels) or an estimation task (**lower** panels) with the two tasks tested in separated sessions. Each task was carried out with two different classes of stimuli either presented sequentially (sequence of flashes shown in the **right**) or simultaneously (arrays of dots, shown on the **left**). Independently from the type of stimulus used, during the discrimination task (**top** row), a test and a reference stimulus was presented sequentially and participants had to indicate which of the two stimuli was more numerous. In the estimation task (**bottom** row), the participants saw only one stimulus that could appear either on the left or right of the screen and their task was to estimate the stimulus numerosity using a virtual numeric keypad.

In this task (Figure 1, top row), a test and a reference stimulus were presented sequentially during each trial on the two sides of the screen (left and right) centered on the screen midline and with a horizontal eccentricity of 12° from the central fixation point. The inter-stimulus interval between the 2 stimuli was 500 ms, and their order was randomized across trials, with the first stimulus (either the test or the reference) always appearing on the left side of the screen. At the end of each trial, the participants were asked to report which stimulus was more numerous (2-alternative forced-choice) by pressing the appropriate key (1 or 2, corresponding to left or right, respectively) on a standard keyboard. The test and reference stimuli could be either a sequence of briefly presented discs or two arrays of dots, with the two conditions tested in separated sessions. Each participant who took part in this task completed 65 trials for each condition.

#### 2.3.2. Estimation Task

In this task (Figure 1, bottom row), a single stimulus was presented on the screen either to the left or right of the central fixation point (position randomized across trials) with a horizontal eccentricity of 12°. After the stimulus presentation (2 s), the participants were required to estimate the stimulus numerosity by typing the number on a virtual keypad appearing on the screen. Participants who took part in this task completed 75 trials for each class of stimuli (sequence of flashes and array of dots).

During all test conditions (for both tasks of discrimination and estimation), no feedback was provided.

### 2.4. Data Analysis

**Figure 2:**
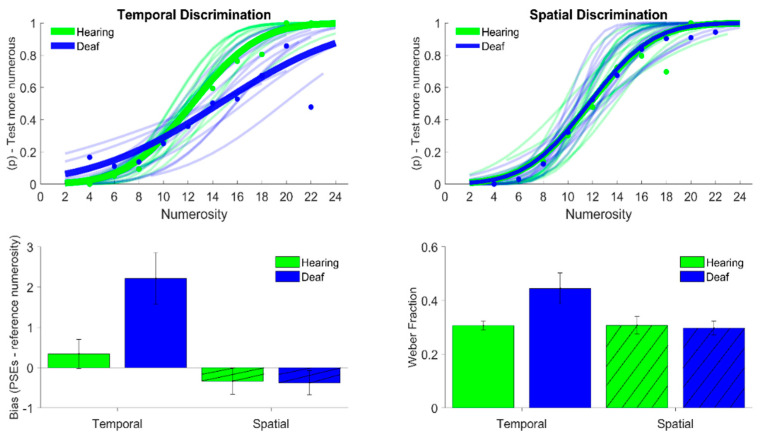
The top panels show the psychometric functions for the discrimination task for both temporal (on the **left**) and spatial (on the **right**) numerosity. The functions represent the cumulative Gaussian (psychometric) fits to the proportion of trials in which participants judged the test stimulus as more numerous against the difference in numerosity between the test and the reference. The bold data points and curves indicate the average psychometric fit across participants, while light colors indicate single-subject data. The bottom-left panel indicates the average bias, defined as the difference between the PSE and reference values, while the bars on the bottom right panel indicate the average Weber fractions. Both for the bias and the Weber fraction, the plain bars indicate the temporal task, while the hatched bars indicate the spatial task. Finally, data for hearing participants are shown in green, while data for the deaf group are shown in blue. Error bars represent ±1 SEM.

For each participant in the discrimination task, we calculated the best-fitting psychometric function to the data. This function shows the probability of the participants to judge the test stimulus to be more numerous than the reference stimulus (Figure 2, top row). Accuracy is represented by the point of subjective equality (PSE), defined as the test numerosity that yields 50% “test more numerous” responses. By subtracting the reference stimulus (12 dots/flash) from the PSE, we obtained the bias measure, defined as the difference between the test stimulus numerosity that was perceived as being as numerous as the reference and the physical numerosity of the latter. A positive value for the bias indicates that the participants showed a tendency to underestimate the numerosity of the test stimulus, while a negative value indicates a perceptual overestimation. Moreover, from the slope of the best fitting psychometric functions, we obtained an estimate of the participants’ precision. Precision was defined in terms of the Weber fraction (i.e., the standard deviation (SD) normalized by the PSE), a dimensionless index of precision that allows the comparison and averaging of performance across different numerosities.

In the estimation task, we computed the average numerical estimates of each participant for each numerosity. The catch trials (i.e., 3 and 4) were excluded from data analysis to avoid including numbers within the subitizing range (see [45,46]), in which numerosity estimates are usually errorless. Then, for each participant, we calculated the average bias by subtracting the average perceived numerosity from the actual physical value and averaging this value across all numerosities. Additionally, for this task, the bias reflected the accuracy of the performance. In the estimation task, the average Weber fraction (WF) was obtained for each participant by dividing the average standard deviation of the numerical estimates across the range by the average numerosity of the range.

We used the same statistical analysis for both tasks, running a linear mixed model analysis (via the “*lme4*” 1.1-21 package in R). We defined GROUP (deaf and hearing participants) and STIMULI (temporal and spatial numerosities) as fixed effects, while the participant was added as a random effect [47]. In addition, since the participants’ GENDER was not balanced across groups for each condition and task, it was added as a random effect to assess its influence on the results. Then, we ran a Wald chi-square test on the linear mixed model (R function Anova, “*car*” package in R). Further statistical analyses were performed via a series of post hoc pairwise tests (“*emmeans*” version 1.5.3 package in R) to obtain an estimate of the marginal means for factor combinations in the model. This analysis allowed us to compute and contrast the predicted probability distributions relative to each response level. We considered pairwise comparisons with *p* < 0.05 to be significant only after applying a family-wise error rate correction (FWER) according to the Holms correction for multiple comparisons. In addition, since the age between participants in the deaf group and hearing controls differed, we tested the correlation between the participants’ ages and the dependent variables of the discrimination task (PSE and JND) to assess whether the difference found between the two groups could be due to age difference. None of these correlations were significant. The results are shown in Table 3.

#### 2.4.1. Discrimination Task

The results for the discrimination task are presented in Figure 2. The top panels show the results for numerosity discrimination of the stimuli presented sequentially (temporal numerosity, left) and simultaneously (spatial numerosity, right) for both classes of participants: deaf (in blue) and hearing (in green). In both conditions, the best-fitting cumulative Gaussian functions represented the percentage of responses where “test more numerous” was perceived as a function of the test numerosity. In the spatial numerosity condition (top right panel), the best fitting psychometric functions to the averaged data (bold curves and data points) almost overlapped to indicate that the performance in numerosity discrimination was rather identical for the two groups of participants. On the contrary, when the participants were required to discriminate the numerosity of sequences of visual stimuli (top left panel), the average psychometric function for the deaf participants was slightly shifted to the right. While the small rightward shift indicates that the deaf group showed a tendency to perceive the variable stimulus as being less numerous than the reference when their physical numerosity was equated, the robust difference in the slope of the curve indicates that the deaf participants required a substantially larger variation in numerosity between the test and reference stimuli to consistently discriminate them as different.

The bar graph at the bottom of Figure 2 indicates the averaged BIAS and Weber fractions (WFs) for both groups of participants in both experimental conditions (spatial and temporal numerosities). The bottom-left panel indicates the average bias for numerosity discrimination, defined as the difference of the averaged BIAS from the actual numerosity of the reference stimulus. Overall, the bias was very close to zero in all the conditions, except when the deaf participants were required to discriminate the numerosity of the stimuli presented sequentially (temporal numerosity). The outcome of the linear mixed model performed on the BIAS showed a significant effect for the stimuli (χ^2^ = 22.61, *p* < 0.01) and a significant interaction between the stimuli and group (χ^2^ = 8.76, *p* < 0.01). Post hoc analysis showed a significant difference only for the deaf group between the two stimuli conditions (t.ratio = 5.32, *p* < 0.01) and between the hearing and deaf group in the temporal stimuli condition (t.ratio = 3.11, *p* < 0.05).

Precision in the numerosity discrimination task was instead assessed in terms of WFs (bottom-right panel of Figure 2). In the condition in which participants had to discriminate the numerosity of arrays of dots (spatial numerosity), the deaf participants (plain blue bar) showed WF values equal to 0.298, a value almost identical to that of the hearing controls (plain green bar; WF = 0.31). However, the deaf participants were significantly less precise in the condition of sequential stimuli (temporal numerosity) compared with the control group, with a difference of approximately 30%. In line with that, the result of a linear mixed model performed on WFs showed a significant effect of the stimuli (χ^2^ = 4.31, *p* < 0.05) and a significant interaction between the stimuli and group (χ^2^ = 4.29, *p* < 0.05). Post hoc tests showed a significant difference only for the deaf group between the two stimuli conditions (t.ratio = 2.87, *p* < 0.05) and between the hearing and deaf group in the temporal stimuli condition (t.ratio = 2.66, *p* < 0.05).

#### 2.4.2. Estimation Task

**Figure 3:**
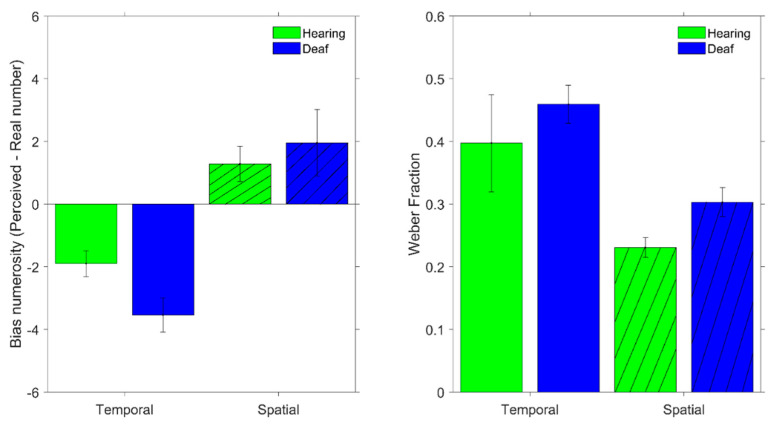
Results of the estimation task in terms of accuracy (**left**) and precision (**right**) for both groups of participants. Data for deaf participants are in blue, while those for the hearing control group are in green. Plain and hatched bars indicate the results for the estimation of the temporal and spatial numerosities, respectively. Error bars represent ±1 SEM.

In a second experiment, the participants were required to estimate the numerosity of either an array of dots or sequences of flashes (tested in separated sessions). Figure 3 shows the results for the estimation task for both groups of participants averaged across all the numerosities tested. The left panel shows the average bias in the numerosity estimates, defined as the difference between the perceived and the actual numerosity averaged across all numerosities and participants. While the two groups of participants showed rather small and positive biases for the estimation of spatial numerosity, the hearing and deaf participants showed an opposite tendency to underestimate the perceived numerosity of the stimuli presented sequentially (negative bias), with such a trend being slightly more pronounced in the deaf group. However, as shown by the linear mixed model, neither the effect of the group (χ^2^ = 0.76, *p* = 0.38) nor the interaction between the group and stimuli (χ^2^ = 2.31, *p* = 0.13) were statistically significant, while we found a significant difference between the temporal and spatial estimation conditions (χ^2^ = 45.23, *p* < 0.01).

The results for the average participant’s precision in the estimation task are shown in the right panel of Figure 3. The Weber fractions for the estimate of the temporal numerosity (stimuli presented sequentially) turned out to be higher than for the spatial numerosity. This result indicates that the internal mapping of the numerosity of the stimuli presented across time was noisier than for the estimate of the numerosity of stimuli presented simultaneously over a given region of space. However, regardless of the kind of stimuli used during the estimation task, the average Weber fraction across the two groups was similar. These results are supported by the statistical results of the linear mixed model, which revealed a significant effect for the stimuli (χ^2^ = 14.39, *p* < 0.01) but not for the group (χ^2^ = 2.40, *p* = 0.12) and no interaction between the group and stimuli (χ^2^ = 0.016, *p* = 0.89).

## 3. Discussion

In the present study, we compared the ability of deaf and hearing participants to perceive numerosity in two different types of tasks (discrimination and estimation) and two different presentation formats (temporal and spatial numerosity). The main goal of the study was to assess whether early deprivation of auditory experience might induce impairment in the processing of numerosity. Such a hypothesis arises from previous studies showing that the precision of temporal processing is affected by early deafness [35,36,37,38,39,40,41,42]. On the other hand, according to the ATOM theory [21], space, time, and numerosity might be processed via a shared, generalized mechanism. If this is the case, it might be expected that the impairment showed in the temporal dimension in deaf individuals could also transfer to numerosity perception, at least in the case in which the numerical magnitude is presented over time (temporal numerosity).

We achieved two main results. First, when comparing the performance of the deaf and hearing individuals in the discrimination task, we found that both the precision and accuracy of the deaf group were worse compared with the control group but only when the stimuli were presented over time (temporal numerosity). On the contrary, when the stimuli were presented simultaneously (spatial numerosity), the performance was similar in the two groups. This result suggests that the numerosity ability of deaf people is strictly dependent on the presentation format of the stimuli used. The second result concerns the different patterns of results obtained in the discrimination and the estimation tasks. We found that the impaired performance in judging sequentially presented stimuli was specifically for the discrimination task, suggesting that the difference in numerosity perception between the groups was task-specific. Therefore, we might speculate that the impairment caused by early hearing deprivation is limited to specific aspects of numerosity perception more closely related to temporal processing, and this, in turn, supports the idea that numerosity perception is not supported by a single mechanism but more likely by different, potentially independent mechanisms dedicated to “spatial” and “temporal” numerosity.

How can we explain this pattern of results? A possibility is that the difference found in the discrimination task between hearing and deaf people, which was selective for the temporal numerosity, may be due merely to the working memory load. More specifically, in the condition with sequential stimuli, the cognitive load could be greater than in the simultaneous condition. Indeed, in the temporal numerosity format, the duration of the stimuli presented was longer, and this might have led to a greater cognitive effort and worse performance compared with the spatial numerosity format. However, we calculated the proportion of correct responses, and previous studies have shown that deaf participants have equal if not superior working memory performance compared with hearing groups [48,49,50]. Finally, for the discrimination task, we calculated the proportion of correct responses (see Supplementary Materials) by distinguishing between trials in which the test stimulus had high or low numerosities, and we found no difference when comparing these two subsets of stimuli. By doing so, we verified that the different performance found in the discrimination task in the sequential stimuli condition for the group of deaf people tapped into a different way of processing the numerosity information, and this was not a problem strictly related to working memory processing. Another possible explanation to account for the present results is to hypothesize an impairment of deaf individuals in the processing of numerical information presented over time, as it would be selectively related to the encoding of temporal (but not spatial) information.

First, it has to be noted that most of the deaf participants tested in this study had previously been tested in an experiment from our group aimed to assess their ability to process temporal information. In the study by Amadeo et al. [35], the participants were tested in a visual temporal bisection task wherein spatio-temporal information in conflict and not in conflict was delivered. The authors observed that when the deaf participants could only leverage temporal cues to accomplish the task, their performance was poorer compared with the hearing controls, with such differences vanishing when spatial cues were provided. The authors concluded that deaf people are unable to correctly process temporal information, and thus, when available, they rely on spatial cues.

Such impairment in temporal processing by deaf individuals could be accounted for in terms of the cross-sensory calibration theory. According to this theory, different sensory modalities calibrate the others during development based on their sensitivity in a specific domain. For instance, vision’s superior spatial resolution contributes to developing spatial processing in audition [51]. The absence of vision during development (i.e., congenitally blind individuals) could cause impairment in complex spatial abilities [33,52]. Instead, the absence of hearing during development (i.e., congenital deafness), due to its superior temporal sensitivity [28,31,34], could halt the calibration of some aspects of temporal perception [35,37,38,39,40,41,42]. It is possible that such impairment is not only limited to temporal processing itself but could also impact other domains related to temporal perception [53], like sequential numerosity. In our experiment, when the numerosity information needed to be extracted from a series of events (temporal presentation), we observed a higher bias and poorer precision in the numerosity judgment. Conversely, when the stimuli did not involve any temporal information, like in the spatial numerosity presentation, the deaf participants had the same performance as the hearing control. Finally, the lack of difference between the spatial and temporal numerosities in the estimation task suggests the involvement of a later, high-level mechanism abstracted from early sensory processing. Note however that while the reduced precision of the deaf participants observed when discriminating temporal numerosities could be interpreted as a noisier representation of numerosity, the directionality of the bias (i.e., overestimation of the reference or underestimation of the test) was more difficult to interpret. Irrespective of this, the bias shows that aside from the increased noise in numerosity representation, the deaf participants were also prone to systematic biases when comparing two stimuli.

In light of this, we propose that the processing of numerosity information might be divided into two steps: first a low-level processing stage with independent subsystems for spatial and temporal numerosity [18,20,21] and then higher-level processing with a generalized numerosity system [14].

These findings are also in line also with recent results showing that brain responses tuned to numerosity could be observed across multiple areas along the dorsal visual stream, from the early visual areas (V1–V3) to higher-order associative areas in the parietal cortex [54,55,56,57,58]. In this scenario, we could hypothesize that the discrimination task could rely more on the first low-level processing stage, and for this reason, it could be more easily affected by early auditory deprivation. Conversely, in the estimation task, approximate numerical information could rely on a supramodal high-level representation of numerosity for which a compensation network for early hearing loss is present [54,56,57,59]. Namely, the specific neural computations entailed by the estimation task, involving a conversion of a perceptual representation into a symbolic one, may trigger a more generalized system abstracted from low-level sensory information and, presumably, able to compensate for different levels of sensory noise affecting perceptual processing.

By focusing exclusively on the ability of deaf individuals to process numerical information, several studies have shown that their numerical (abstract and symbolic) processing abilities are equal to those of hearing people [60,61,62,63], although deaf people appear to be slower to access such information [64,65,66]. These studies considered numerosity estimation in which only spatial numerical tasks were performed. Moreover, most of these studies are confined to the subitizing domain. For example, Bull et al. [60], analyzing the distance effect and the spatial numerical association of the response codes effect, found comparable performance between the hearing and deaf groups, as well as similar performance in a subitizing task.

To the best of our knowledge, only one previous article assessed the dissociation between temporal and spatial numerosity in deaf children, but only relative to the subitizing domain. The authors found no differences in performance between the deaf and hearing children in either the spatial or temporal number task [63]. Thus, our study is the only one that attempted to disentangle temporal and spatial numerosity information in adults in the range of numerosity when comparing deaf and hearing individuals. Nevertheless, future experiments would be interesting to understand whether higher-level processes are sensitive to a more generalized ability for symbolic representation.

Overall, our results suggest that the numerosity processing mechanism was neither entirely composed by multiple systems nor completely generalized but likely organized in a hierarchical fashion of increasing complexity. The absence of early auditory experience, as in deaf individuals, may affect the processing of temporally defined numerosity at the lower step of this hierarchy. However, downstream to sensory processing, the system is likely to compensate for such an impairment when participants are engaged in tasks entailing a more abstract mapping of numerosity information. This can be linked to the ATOM theory [21], for which space, time, and numerosity might be processed via shared mechanisms. Nevertheless, since our behavioral data did not allow us to draw a firm conclusion on this point, further investigations at the neural level are needed to test this hypothesis.

Finally, we need to acknowledge two possible limitations of the present study: the potential confound of non-numerical dimensions in the numerosity tasks employed and the co-varying cognitive contributors to numerical processing.

For the first point, when it comes to simultaneously presented arrays of dots, other stimulus dimensions like density or the total area covered by the dots could indeed contribute to participants’ judgments, especially in a discrimination task. Although this topic is currently debated in the numerosity perception literature (see, for instance [67]), there is evidence that numerosity represents the most salient information extracted from dot arrays [9,68,69]. When it comes to sequential numerosity, on the other hand, the stimuli used in the present study are potentially confounded by the temporal frequency (or rate), which co-varied with the numerosity. Indeed, since we kept the total duration of the sequences constant (i.e., 2 s), the higher the numerosity, the higher the temporal frequency of the sequence. However, also in this context, previous studies have shown that numerosity is systematically extracted and processed even when other dimensions could interfere with it. This was demonstrated by measuring what stimulus dimensions drove perceptual effects such as serial dependence [70] and adaptation [71]. In both cases, the data suggest that the representation of a sequence is more strongly based on its numerosity rather than the duration or temporal frequency, at least when participants are actively attending to numerosity. However, since we did not directly control for this aspect of stimuli, the potential role of other stimulus dimensions in the present results remains an open question that should be addressed by future studies.

Regarding the second limitation, it has been shown that in deaf adolescents, the processing of numerical magnitude is associated with arithmetic, even when controlling for general cognitive abilities [72]. However, this difference seems to be mainly related to the use of non-symbolic and symbolic stimuli, which in the latter case makes processing numerosity more challenging for deaf people than their hearing peers, especially in children (for a review see [73]; [74,75]). In the present study, this discriminant was controlled for in the use of non-symbolic stimuli. Furthermore, in a previous study in which our own stimuli were used, there appeared to be no correlation between math scores and sensitivity in temporal or spatial numerosity tasks [18]. Nevertheless, we believe that in future studies, the cognitive component should be taken into account as a variable that may influence numerosity processing, especially in studies involving children.

## 4. Conclusions

To conclude, our results show the impact of deafness on approximate temporal numerical abilities. Indeed, the absence of cross-sensory calibration from audition to vision impairs the processing of sequential and temporal numerosity, especially when the task requires a relative judgment relying more on perceptual processing. These results highlight the flexibility of the numerosity processing system and its hierarchical organization, showing that high-level processing could overcome a low-level deficit, referring to an abstract representation of magnitude.

## Figures and Tables

**Table 1:** Information about the sample size and average age of participants for each task for both deaf and control groups. SQDT = sequential discrimination task; SMDT = simultaneous discrimination task; SQET = sequential estimation task; SMET = simultaneous estimation task.

Task	Deaf Group	Control Group
SQDT	n = 13, 3 males; mean age: 44.07 ± 17.1 years	n = 13, 7 males; mean age: 29.85 ± 4.16 years
SMDT	n = 14, 3 males; ages: 38.78 ± 16.6 years	n = 14, 7 males; mean age: 29.77 ± 4 years
SQET	n = 14, 1 male; mean age: 36 ± 13.2 years	n = 14, 7 males; mean age: 31.28 ± 3.24 years
SMET	n = 14, 3 males; mean age: 38 ± 14.8 years	n = 14, 7 males; mean age: 31.28 ± 3.24 years

**Table 2:** Demographic information about deaf participants and type of task performed. SQDT = sequential discrimination task; SMDT = simultaneous discrimination task; SQET = sequential estimation task; SMET = simultaneous estimation task.

Participant	Age	Gender	SQDT Sequential Discrimination Task	SMDT Simultaneous Discrimination Task	Sequential Estimation Task (SQET)	Simultaneous Estimation Task (SMET)	Detection of Deafness	Age at First Exposure to Italian Sign Language (in Years)
S03	57	F	X	X		X	2–4 y.a.	26
S05	25	F	X	X			2–4 y.a.	11
S08	25	F	X	X	X	X	1–2 y.a.	15
S10	61	M	X	X		X	birth	native
S11	38	F	X	X	X		4 y.a.	13
S12	30	F	X	X	X	X	2–4 y.a.	6
S16	22	F	X	X	X		2–4 y.a.	native
S22	32	M	X	X			1–2 y.a.	native
S33	56	F	X		X		1–2 y.a.	12
S30	57	F	X	X	X		1–2 y.a.	30
S31	72	M	X				birth	5
S32	62	F	X		X		birth	15
S04	34	F		X	X	X	birth	native
S09	24	F		X	X	X	birth	native
S14	26	F		X	X	X	birth	native
S15	38	F		X	X	X	1 y.a.	native
S18	74	M		X			4–5 y.a.	7
S07	28	F			X	X	4–5 y.a.	18
S21	28	M			X	X	birth	25
S34	36	F	X		X		birth	21
S01	42	M				X	2–4 y.a.	native
S02	34	F				X	birth	6
S17	67	F				X	birth	native

**Table 3:** Correlations results between the ages of the participants and the JND or PSE of discrimination task 3: RESULTS.

		AGE	
		Pearson’s r	*p*-Value
Temporal condition	PSE deaf	0.102	0.74
	WF deaf	0.13	0.66
	PSE hearing	0.102	0.74
	WF hearing	0.33	0.24
Spatial condition	PSE deaf	0.33	0.24
	WF deaf	0.38	0.17
	PSE hearing	0.47	0.09
	WF hearing	0.17	0.55

## Data Availability

The data will be available upon request. The data and materials for all experiments will be available upon request.

## Supplementary Materials

The following supporting information can be downloaded at: https://www.mdpi.com/article/10.3390/brainsci12020179/s1, Table S1: Participant comparison with study by Amadeo and colleagues (2019).

## References

[1] Dehaene S., Brannon E. (2011). Space, Time and Number in the Brain: Searching for the Foundations of Mathematical Thought.

[2] Dehaene S., Dehaene-Lambertz G., Cohen L. (1998). Abstract representations of numbers in the animal and human brain. Trends Neurosci..

[3] Nieder A. (2002). Representation of the Quantity of Visual Items in the Primate Prefrontal Cortex. Science.

[4] Nieder A. (2012). Supramodal numerosity selectivity of neurons in primate prefrontal and posterior parietal cortices. Proc. Natl. Acad. Sci. USA.

[5] Nieder A. (2016). The neuronal code for number. Nat. Rev. Neurosci..

[6] Nieder A., Diester I., Tudusciuc O. (2006). Temporal and Spatial Enumeration Processes in the Primate Parietal Cortex. Science.

[7] Nieder A., Miller E.K. (2004). A parieto-frontal network for visual numerical information in the monkey. Proc. Natl. Acad. Sci. USA.

[8] Castaldi E., Aagten-Murphy D., Tosetti M., Burr D., Morrone M.C. (2016). Effects of adaptation on numerosity decoding in the human brain. NeuroImage.

[9] Fornaciai M., Brannon E.M., Woldorff M.G., Park J. (2017). Numerosity processing in early visual cortex. NeuroImage.

[10] Piazza M., Izard V. (2009). How Humans Count: Numerosity and the Parietal Cortex. Neuroscientist.

[11] Piazza M., Izard V., Pinel P., Le Bihan D., Dehaene S. (2004). Tuning Curves for Approximate Numerosity in the Human Intraparietal Sulcus. Neuron.

[12] Piazza M., Mechelli A., Price C.J., Butterworth B. (2006). Exact and approximate judgements of visual and auditory numerosity: An fMRI study. Brain Res..

[13] Burr D., Ross J. (2008). A Visual Sense of Number. Curr. Biol..

[14] Arrighi R., Togoli I., Burr D.C. (2014). A generalized sense of number. Proc. R. Soc. B Biol. Sci..

[15] Anobile G., Cicchini G.M., Burr D.C. (2016). Number as a Primary Perceptual Attribute: A Review. Perception.

[16] Anobile G., Domenici N., Togoli I., Burr D., Arrighi R. (2020). Distortions of visual time induced by motor adaptation. J. Exp. Psychol. Gen..

[17] Maldonado Moscoso P.A., Cicchini G.M., Arrighi R., Burr D.C. (2020). Adaptation to hand-tapping affects sensory processing of numerosity directly: Evidence from reaction times and confidence. Proc. R. Soc. B Biol. Sci..

[18] Anobile G., Arrighi R., Castaldi E., Grassi E., Pedonese L., Moscoso P.A.M., Burr D.C. (2018). Spatial but not temporal numerosity thresholds correlate with formal math skills in children. Dev. Psychol..

[19] Cavdaroglu S., Katz C., Knops A. (2015). Dissociating estimation from comparison and response eliminates parietal involvement in sequential numerosity perception. NeuroImage.

[20] Cavdaroglu S., Knops A. (2019). Evidence for a Posterior Parietal Cortex Contribution to Spatial but not Temporal Numerosity Perception. Cereb. Cortex.

[21] Walsh V. (2003). A theory of magnitude: Common cortical metrics of time, space and quantity. Trends Cogn. Sci..

[22] Fornaciai M., Togoli I., Arrighi R. (2018). Motion-induced compression of perceived numerosity. Sci. Rep..

[23] Xuan B., Chen X.-C., He S., Zhang D.-R. (2009). Numerical magnitude modulates temporal comparison: An ERP study. Brain Res..

[24] Xuan B., Zhang D., He S., Chen X. (2007). Larger stimuli are judged to last longer. J. Vis..

[25] Roitman J., Brannon E. (2003). Nonverbal Representations of Time and Number in Animals and Human Infants. Functional and Neural Mechanisms of Interval Timing.

[26] Bonato M., Zorzi M., Umiltà C. (2012). When time is space: Evidence for a mental time line. Neurosci. Biobehav. Rev..

[27] Eimer M. (2004). Multisensory Integration: How Visual Experience Shapes Spatial Perception. Curr. Biol..

[28] Burr D., Banks M.S., Morrone M.C. (2009). Auditory dominance over vision in the perception of interval duration. Exp. Brain Res..

[29] Burr D., Gori M. (2012). Multisensory integration develops late in humans. The Neural Bases of Multisensory Processes.

[30] Gori M. (2015). Multisensory Integration and Calibration in Children and Adults with and without Sensory and Motor Disabilities. Multisens. Res..

[31] Gori M., Sandini G., Burr D. (2012). Development of Visuo-Auditory Integration in Space and Time. Front. Integr. Neurosci..

[32] Pasqualotto A., Proulx M.J. (2012). The role of visual experience for the neural basis of spatial cognition. Neurosci. Biobehav. Rev..

[33] Gori M., Sandini G., Martinoli C., Burr D.C. (2014). Impairment of auditory spatial localization in congenitally blind human subjects. Brain.

[34] Recanzone G.H. (2003). Auditory Influences on Visual Temporal Rate Perception. J. Neurophysiol..

[35] Amadeo M.B., Campus C., Pavani F., Gori M. (2019). Spatial Cues Influence Time Estimations in Deaf Individuals. IScience.

[36] Amadeo M.B., Tonelli A., Campus C., Gori M. (2022). Reduced flash lag illusion in early deaf individuals. Brain Res..

[37] Bolognini N., Cecchetto C., Geraci C., Maravita A., Pascual-Leone A., Papagno C. (2012). Hearing Shapes Our Perception of Time: Temporal Discrimination of Tactile Stimuli in Deaf People. J. Cogn. Neurosci..

[38] Gori M., Chilosi A., Forli F., Burr D. (2017). Audio-visual temporal perception in children with restored hearing. Neuropsychologia.

[39] Heming J.E., Brown L.N. (2005). Sensory Temporal PROCESSING in adults with Early Hearing Loss. Brain Cogn..

[40] Kowalska J., Szelag E. (2006). The effect of congenital deafness on duration judgment. J. Child Psychol. Psychiatry.

[41] Rileigh K.K., Odom P.B. (1972). Perception of rhythm by subjects with normal and deficient hearing. Dev. Psychol..

[42] Sterritt G.M., Camp B.W., Lipman B.S. (1966). Effect of early auditory deprivation upon auditory and visual information processing. Percept. Mot. Ski..

[43] Kumle L., Võ M.L.-H., Draschkow D. (2021). Estimating power in (generalized) linear mixed models: An open introduction and tutorial in R. Behav. Res. Methods.

[44] Watson A.B., Pelli D.G. (1983). QUEST: A Bayesian adaptive psychometric method. Percept. Psychophys..

[45] Anobile G., Arrighi R., Burr D.C. (2019). Simultaneous and sequential subitizing are separate systems, and neither predicts math abilities. J. Exp. Child Psychol..

[46] Kaufman E.L., Lord M.W., Reese T.W., Volkmann J. (1949). The Discrimination of Visual Number. Am. J. Psychol..

[47] Cnaan A., Laird N.M., Slasor P. (1997). Using the general linear mixed model to analyse unbalanced repeated measures and longitudinal data. Stat. Med..

[48] Arnold P., Murray C. (1998). Memory for faces and objects by deaf and hearing signers and hearing nonsigners. J. Psycholinguist. Res..

[49] Bavelier D., Dye M.W.G., Hauser P.C. (2006). Do deaf individuals see better?. Trends Cogn. Sci..

[50] Cattani A., Clibbens J., Perfect T.J. (2007). Visual memory for shapes in deaf signers and nonsigners and in hearing signers and nonsigners: Atypical lateralization and enhancement. Neuropsychology.

[51] Alais D., Burr D. (2004). Ventriloquist Effect Results from Near-Optimal Bimodal Integration. Curr. Biol..

[52] Lewald J. (2002). Vertical sound localization in blind humans. Neuropsychologia.

[53] Kral A., O’Donoghue G.M. (2010). Profound Deafness in Childhood. N. Engl. J. Med..

[54] Castaldi E., Vignaud A., Eger E. (2019). Mapping numerical perception and operations in relation to functional and anatomical landmarks of human parietal cortex. BioRxiv.

[55] Fornaciai M., Park J. (2018). Early Numerosity Encoding in Visual Cortex Is Not Sufficient for the Representation of Numerical Magnitude. J. Cogn. Neurosci..

[56] Harvey B.M., Dumoulin S.O. (2017). A network of topographic numerosity maps in human association cortex. Nat. Hum. Behav..

[57] Roggeman C., Santens S., Fias W., Verguts T. (2011). Stages of Nonsymbolic Number Processing in Occipitoparietal Cortex Disentangled by fMRI Adaptation. J. Neurosci..

[58] Van Rinsveld A., Guillaume M., Kohler P.J., Schiltz C., Gevers W., Content A. (2020). The neural signature of numerosity by separating numerical and continuous magnitude extraction in visual cortex with frequency-tagged EEG. Proc. Natl. Acad. Sci. USA.

[59] Andin J., Fransson P., Rönnberg J., Rudner M. (2018). fMRI Evidence of Magnitude Manipulation during Numerical Order Processing in Congenitally Deaf Signers. Neural Plast..

[60] Bull R. (2006). Subitizing, Magnitude Representation, and Magnitude Retrieval in Deaf and Hearing Adults. J. Deaf. Stud. Deaf. Educ..

[61] Bull R. (2008). Deafness, numerical cognition, and mathematics. Deaf Cognition: Foundations and Outcomes.

[62] Epstein K.I., Hillegeist E.G., Grafman J. (1994). Number processing in deaf college students. Am. Ann. Deaf..

[63] Zarfaty Y. (2004). The Performance of Young Deaf Children in Spatial and Temporal Number Tasks. J. Deaf. Stud. Deaf. Educ..

[64] Bull R., Marschark M., Blatto-Vallee G. (2005). SNARC hunting: Examining number representation in deaf students. Learn. Individ. Differ..

[65] Bull R., Marschark M., Sapere P., Davidson W.A., Murphy D., Nordmann E. (2011). Numerical estimation in deaf and hearing adults. Learn. Individ. Differ..

[66] Chinello A., de Hevia M.D., Geraci C., Girelli L. (2012). Finding the spatial-numerical association of response codes (SNARC) in signed numbers: Notational effects in accessing number representation. Funct. Neurol..

[67] Leibovich T., Katzin N., Harel M., Henik A. (2017). From “sense of number” to “sense of magnitude”: The role of continuous magnitudes in numerical cognition. Behav. Brain Sci..

[68] DeWind N.K., Park J., Woldorff M.G., Brannon E.M. (2019). Numerical encoding in early visual cortex. Cortex.

[69] Park J., DeWind N.K., Woldorff M.G., Brannon E.M. (2015). Rapid and Direct Encoding of Numerosity in the Visual Stream. Cereb. Cortex.

[70] Fornaciai M., Park J. (2019). Serial dependence generalizes across different stimulus formats, but not different sensory modalities. Vis. Res..

[71] Togoli I., Arrighi R. (2021). Evidence for an A-Modal Number Sense: Numerosity Adaptation Generalizes Across Visual, Auditory, and Tactile Stimuli. Front. Hum. Neurosci..

[72] Chen L., Wang Y., Wen H. (2021). Numerical Magnitude Processing in Deaf Adolescents and Its Contribution to Arithmetical Ability. Front. Psychol..

[73] Buyle M., Marlair C., Crollen V. (2021). Blindness and deafness: A window to study the visual and verbal basis of the number sense. Diversity Dimensions in Mathematics and Language Learning.

[74] Masataka N. (2005). Differences in Arithmetic Subtraction of Nonsymbolic Numerosities by Deaf and Hearing Adults. J. Deaf. Stud. Deaf. Educ..

[75] Rodríguez-Santos J.M., Calleja M., García-Orza J., Iza M., Damas J. (2014). Quantity processing in deaf and hard of hearing children: Evidence from symbolic and nonsymbolic comparison tasks. Am. Ann. Deaf..

