# Supplementary material for: Deprivation of Auditory Experience Influences Numerosity Discrimination, but Not Numerosity Estimation
Source: Brain Sci. 2022 Jan 29;12(2):179. doi: 10.3390/brainsci12020179 (PMC8869924; doi:10.3390/brainsci12020179)
Supplement: Supplementary file 1 [file brainsci-12-00179-s001.zip › brainsci-1501888-supplementary.pdf]

Supplementary materials

The role of auditory experience in developing the sense of number

Alessia Tonelli, Irene Togoli, Roberto Arrighi, Monica Gori

Participants

The majority of deaf participants that took part in the experiment have already participated in a previous experiment in our lab by Amadeo et al. (2019) in which were assessed their abilities in a temporal perception task, i.e. temporal bisection task. In this study, it was shown that people with hearing impairments have difficulty in correctly processing temporal information. The following table (STable 1) shows which of our participants also participated in the study by Amadeo et al. The code for participants in that study refers to the table published in the supplementary materials by the authors.

Table S1. Participant comparison with study by Amadeo and colleagues (2019)

Participant present experiment	Age	Participant Amadeo (2019)
S03	56	S01
S05	25	
S08	24	S07
S10	61	S09
S11	38	S08
S12	30	S12
S16	22	S13
S22	32	S16
S33	56	
S30	57	
S31	72	
S32	62	
S04	33	S04
S09	23	S05
S14	26	S11
S15	38	S14
S18	74	S15
S07	28	S06
S21	28	S17
S34	36	

S01	42	S02
S02	34	S03
S17	67	

Moreover, in figure S1 are reported the results of the power analysis for the linear mixed model based on different sample size.

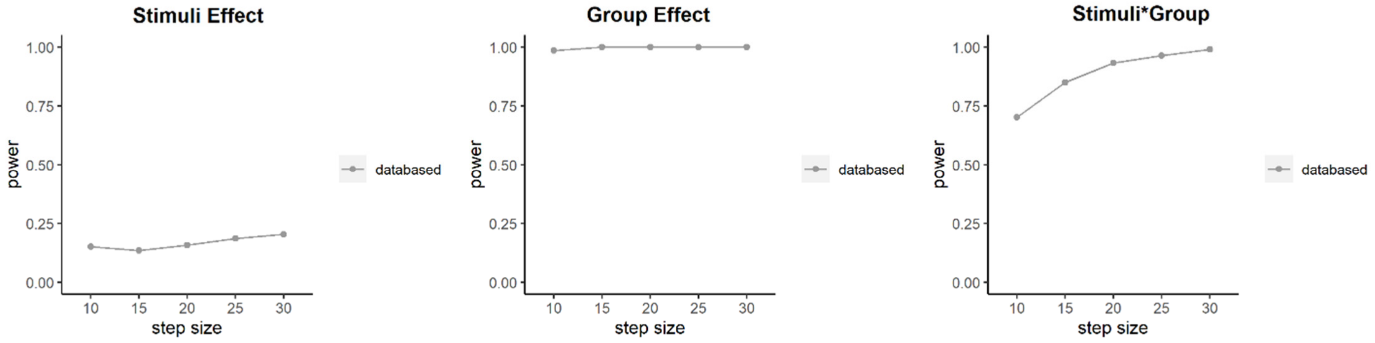

Figure S1. Plots of statistical power distribution versus sample size for each effect and interaction (on the right) for a linear mixed model analysis.

Supplementary statistical analysis

For the discrimination task in both conditions and groups, we analyzed the percentage of correct responses to test whether there might be an influence of mnemonic load in the performance. Specifically, in the sequential (temporal) stimulus condition a high number of flashes of the test stimulus might require greater working memory involvement compared to lower numbers. We calculated the proportion of correct responses using as threshold for low numbers all trials with numbers below nine, and for high numbers all the trials with numbers above fifteen. The results are shown in figure S2 in which there seems to be no difference between the proportion of correct responses for low (in red) and high (in magenta) numbers in both conditions and groups. To get a statistical match, we run four paired two-tails t-test to compare the low and high number sets in each condition of each group. All p-values were Holms corrected for multiple comparisons.

We did not find any statistical difference for anyone of the t-test (control temporal: $t = 1.14$, $p = 0.56$, $d = 0.315$; control spatial: $t = 0.41$, $p = 0.69$, $d = 0.11$; deaf spatial: $t = 1.52$, $p = 0.46$, $d =$

0.42), not even between low and high numbers for the temporal condition in the deaf group (t = 2.075, p = 0.25, *d* = 0.599).

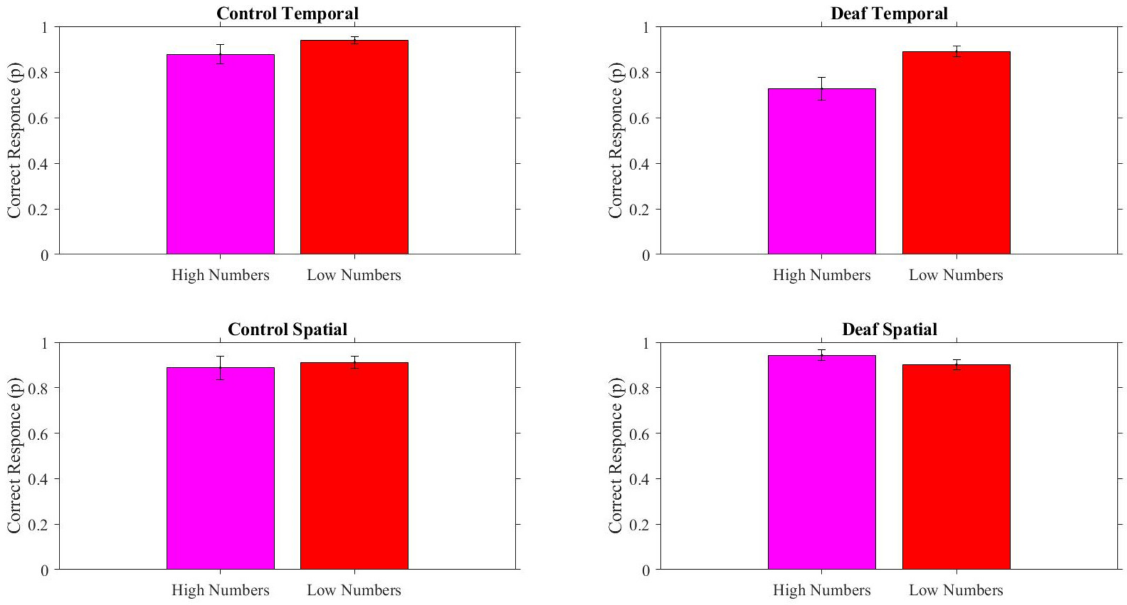

Figure S2. Proportion of correct responses in the discrimination task for both groups and conditions. Magenta's bars represent the proportion of correct responses for the high numbers, while the bars in red are the proportions for the low numbers. Error bars ± 1 SEM.

To check that the result obtained in the main analyses of the average of the estimated values, was not dependent on a greater weight of one of the single numerosities tested rather than the other, , we calculated the Weber Fraction for each numerical quantity included in the main analysis for estimation of precision. Results are shown in figure S3, which it is presents a fluctuation between the numerosity reported (range from 5 to 20). To explain the graphical data, also in this case, we used the linear mixed models (via the "*lme4*" 1.1-21 package in R). We defined GROUP (deaf, hearing) and STIMULI (temporal, spatial) as fixed effects, while participant and numerical range (from 5 to 20) were added as a random effect. Then, we run a Wald chi-square test on the linear mixed model (R function Anova, "c*ar*" package in R). The outcome confirms the results obtained in the main analysis in which all numerosity were averaged, we found a significant effect of stimuli

($\chi2 = 9.62$, $p < 0.01$), but no significance for group ($\chi2 = 0.26$ $p = 0.61$) and interaction ($\chi2 = 2.54$, $p = 0.12$).

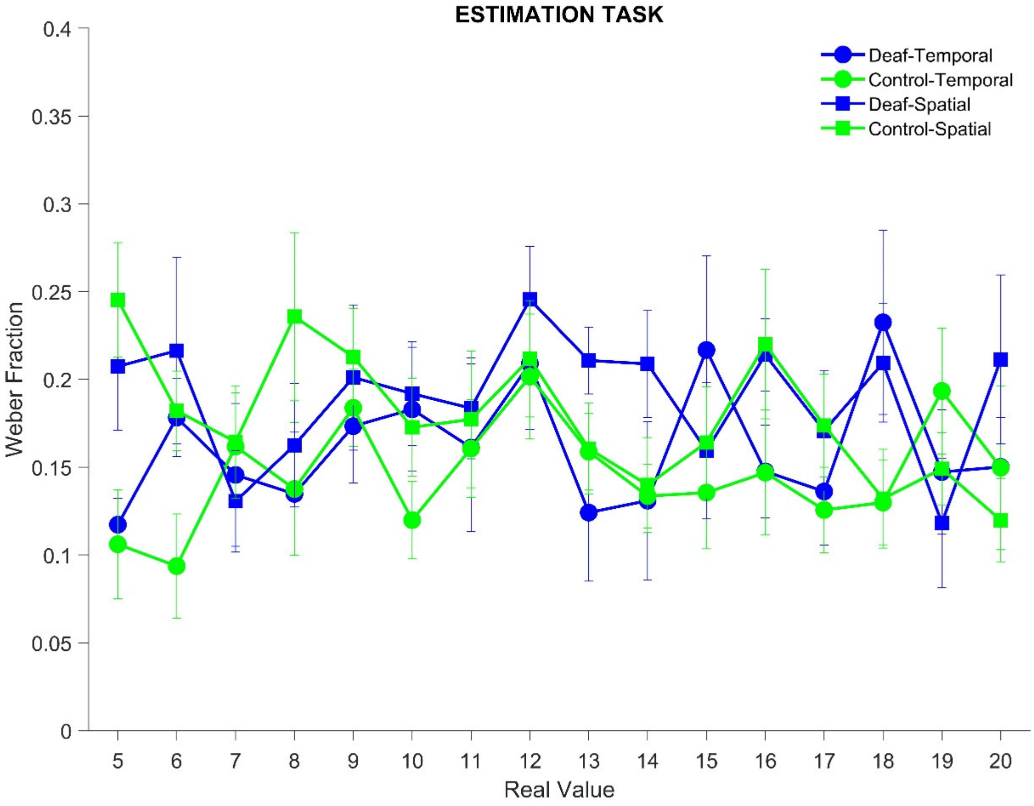

Figure S3. The plot shows the WF for each numerosity tested in the estimation task. The results for the deaf group are in blue, while the one for the hearing group are in green. The shape of the scatters differentiates the results of the two conditions. The circle is used for the temporal type of stimuli, while the squares for the spatial type of stimuli. Error bars ± 1 SEM.

In a recent review regarding math abilities in deaf and hard hearing children (Santos & Cordes, 2021), it has been pointed out that lack of exposure to sign language from the earliest years of life can cause difficulties with numbers and mathematics. Therefore, we correlated the age at which

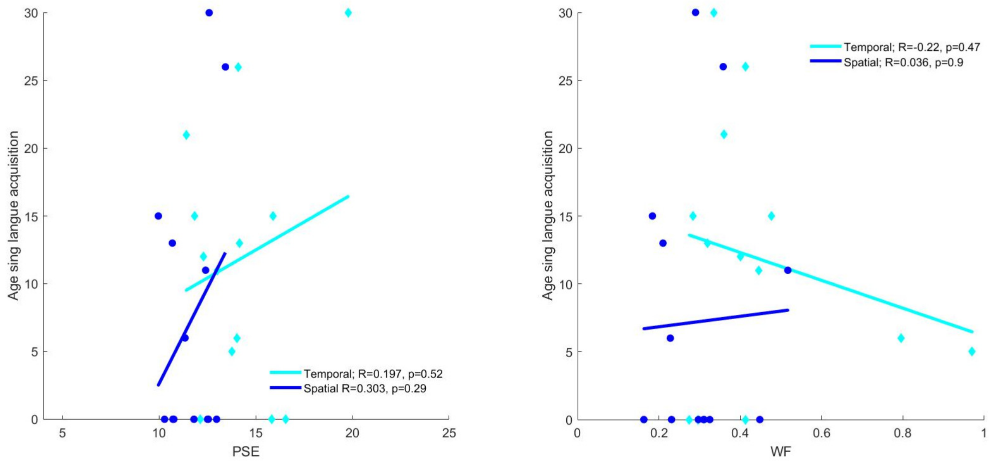

Figure S3. The plots show the correlation between the Age Sign language acquisition (y-axis) and the PSE (on the right) and the WF (on the left). The correlation was calculated separately for the temporal condition (in cyan) and the spatial condition (in blue).

deaf participants acquired sign language with PSE and WF on the discrimination task for both conditions. For native signers we considered the value as zero. No correlation was significant, indicating that in this case early exposure to sign language does not influence the behavior described in the discrimination task.

References

Amadeo, M. B., Campus, C., Pavani, F., & Gori, M. (2019). Spatial Cues Influence Time Estimations in Deaf Individuals. *IScience*, *19*, 369–377. https://doi.org/10.1016/j.isci.2019.07.042

Santos, S., & Cordes, S. (2021). Math abilities in deaf and hard of hearing children: The role of language in developing number concepts. *Psychological Review*. https://doi.org/10.1037/rev0000303
